# Rupture of the Trachea

**Published:** 1844-06

**Authors:** 


*Rupture of the Trachea.*—Dr. Bredscheider reports in *Casper's Wochenschrift*, the case of an infant fifteen months old, affected with bronchitis, who after a spell of coughing, had an emphysematous tumor in front of the neck. A small incision was made in this tumor, which gave issue to air, which escaped with a hissing noise. The child died two hours after the accident, and on examination a rupture half an inch long was found in the trachea, below its first ring.—*Journ. de Connaiss. Méd. Chirurg.*—*Am. Jour. Med. Sci.*

